# Chronic Suppuration of the Middle-Ear and Chronic Empyema of the Nasal Accessory Sinuses

**Published:** 1900-05

**Authors:** Frank Whitehill Hinkel

**Affiliations:** Buffalo, N. Y. Clinical Professor of laryngology, Medical Department, University of Buffalo


### CHRONIC SUPPURATION OF THE MIDDLE-EAR AND CHRONIC EMPYEMA OF THE NASAL ACCESSORY SINUSES.

### REPORT OF TWO CASES WITH OPERATIONS.[1]

By FRANK WHITEHILL HINKEL, A. M., M. D., Buffalo, N. Y.
Clinical Professor of laryngology, Medical Department, University of Buffalo.

### I. Chronic Suppuration of the Middle-Ear, treated by the Stacke Radical Operation Three Weeks Since.

MARY G., aged 12; examined December 27, 1899. Since infancy has had purulent inflammation of the left middle-ear with constant highly offensive discharge. Has never had pain or mastoid symptoms. Is very deaf in left ear. Has had no treatment, except cleansing and boric acid insufflation. A solution of permanganate of potassium has been used daily as a deodorant.

*Examination.*—A highly offensive pus filled the left auditory canal. After its removal there was seen a sodden, markedly funnel-shaped,

1. Read before the Section on Ophthalmology, Laryngology and Otology, Buffalo Academy of Medicine, March 12, 1900.

membrane at the fundus of the auditory canal with a pin-hole perforation at the deepest point. The tissues were very insensitive, and syringing through the small perforation removed cheesy offensive flakes.

January 2, 1900, at the Buffalo General Hospital, under chloroform, the membrane at the fundus of the left auditory canal was removed. No ossicles were found. A quantity of granulation tissue was curetted from the accessible walls of the tympanum. The discharge now became odorless and considerably reduced in quantity, but in spite of careful local treatment there was no further improvement.

On January 25th, I removed a moderate-sized mass of adenoids from the vault of the pharynx and again curetted the middle-ear. The membrane reformed in part, as it had done after the first operation. Tender, easily-bleeding, granulations appeared in the deeper portions of the canal and the discharge, though further diminished, again became stationary as regards quality and quantity.

February 21st, at the Buffalo General Hospital, under chloroform, the radical operation was performed by Stacke's method. After the usual mastoid incision the auricle and membranous auditory canal were separated from their bony attachment and the bony partition between the epitympanum, after thorough curettage of the exposed cavities and the auditory canal, was chiselled away, together with the upper portion of the posterior wall of the auditory canal; the mastoid incision was closed at once and healed kindly. She now hears the watch at four inches in the left ear and low whisper at fifteen feet.

*Remarks.*—As this case had never had thorough surgical treatment by way of the natural passages, I attempted at first to correct the diseased condition by that channel. After the second operation, as the treatment had been thorough and careful for about six weeks without cure, I advised the radical operation. As there had been no evidence of involvement of the mastoid, and it was probably sclerosed (as was found to be the case during the operation) I adopted Stacke's method of entrance to the antrum through the wall of the auditory canal rather than through the mastoid cortex. This involves less scar or depressed cicatrix than occurs in those cases where it is necessary to have free access to the mastoid cells. I have recently operated on a case of colesteatoma of the right ear, in which it was necessary to enter the enlarged antrum through the mastoid cortex by the method of Schwartze, and after removal of the foul mass that had dilated the antrum and tympanum, to remove a considerable part of the anterior and external wall of the sclerosed mastoid process, leaving a large opening behind the ear for access to and treatment of the cavity thus made. As life was at stake, cosmetic effects could not be considered as in the present case. The tenderness which followed the tentative operations upon the case now before you disappeared after the more radical operation. You will notice how slight are the traces of the incision behind the ear. Though some granulations have occurred in the fundus of the wound, its condition is satisfactory, considering the short time that has elapsed since the operation. The general health has already begun to improve and the hearing is distinctly better. These cases often require to be kept under observation for many weeks or months after operating before the required cicatrised or dermoid condition of the walls of the middle-ear occurs.

### II. Chronic Empyema of the Nasal Accessory Sinuses in which the Antrum, Frontal Sinus, Ethmoid Cells, and Sphenoidal Sinus have been Opened.

E. B., aged 21, was first examined June 24, 1898. He gave a history of cough since childhood, much increased the past two years, with purulent expectoration. These symptoms are increased if he lies on his left side or stoops forward. He notices at times a peculiar bitter taste when in this posture. Examination of the chest showed no lesions to account for the symptoms. There was a somewhat polypoid condition of the middle turbinated bodies in each nasal chamber, and much viscid muco-pus in the upper part of the nose and the vault of the pharynx. Trans-illumination was negative, there being darkness over both antrums and both frontal sinuses.

Puncture of the nasal walls of both antrums with Krause's trochar was followed by a discharge of curded odorless pus from each. Cleansing of the antrum by this means was kept up for sometime without permanent improvement. Later I removed the anterior extremities of both middle turbinates and opened both antrums through the canine fossa. At the time of the antral operation no pus was found in either antrum, nor was the lining mucous membrane greatly thickened. The ethmoid cells at different times were broken down by curette and forceps, and drainage secured. The discharge from the right nostril was much improved by the operations, but the left continued about the same, though the cough was improved considerably. The case passed from observation for a time.

In December, 1899, he developed pain and tenderness over the left frontal sinus. Syringing the left antrum, removed considerable pus. The right antrum was found free from pus. The pus continued in the left middle meatus, immediately after the syringing of the antrum.

A diagnosis of frontal empyema being made after careful examination, in January, 1900, at the Buffalo General Hospital, I opened the left frontal sinus through its anterior wall, and also opened the left antrum through its anterior wall and made a large opening connecting the antrum with the inferior meatus, through the nasal wall. I found no pus in either cavity at the time of operation. I am able since to probe easily the fronto-nasal duct. Recovery from both operations was prompt, but the cough and discharge, though modified, continued. The source of pus was now distinctly localised in the upper back part of the left nasal chamber; and under cocaine, with an electric drill and sharp spoon, I made a large opening through its anterior wall into the left sphenoid sinus, washing from it a considerable amount of creamy pus. I curetted carefully the sinus walls so far as accessible. There is great improvement in the night cough, the nasal discharge and the expectoration. The discharge from the sphenoid sinus still continues, but is growing less under treatment.

*Remarks.*—You see how slight a scar is left from the frontal operation. The regrowing eyebrow already largely conceals the cicatrix, and when in time it loses its pinkish color, the scar will scarcely be noticeable. The probe passes easily from the inferior meatus into the antrum through the opening I have made in the antro-nasal wall. I no longer find pus in the antrum or frontal sinus. It is interesting to notice from the history of the case how the maxillary antrum, in which was found pus on experimental puncture, was found empty at the time of operation, as was the frontal sinus. The posture of the patient upon the back during the operation will account for this, these cavities being filled by drainage from the diseased sphenoid and ethmoid cells rather than by secretion from their own mucous membranes. I have elsewhere reported cases of empyema of the antrum dependent upon abscess of the frontal sinus. In this case it may be regarded as dependent upon disease of the sphenoid and ethmoid cells. The case illustrates well the obscurity and difficulty of diagnosis often met with in chronic sinus disease. My method of opening the anterior wall of the sphenoid sinus with the electric drill and sharp spoon, I have used in some half dozen cases with excellent success, both as regards the steps of the operation and benefit to the patient. The difficulty and possible dangers of the operation are obvious and need no comment. I show this case because it is probable that many cases of chronic intractable cough with purulent expectoration have a similar cause. I have several such records upon my case-book. So far as I know, the case is unique in that every nasal accessory sinus has been opened and subjected to treatment.

412 Franklin Street.

---

Montreal is reported to be contemplating the erection of a sanitarium for consumptives. Mount Royal has been suggested as a site, although this location has been objected to on account of its proximity to the city.

